# Supplementary material for: Successful Treatment of Persistent SARS-CoV-2 Infection in a B-Cell Depleted Patient with Activated Cytotoxic T and NK Cells: A Case Report
Source: Int J Mol Sci. 2021 Oct 10;22(20):10934. doi: 10.3390/ijms222010934 (PMC8535727; doi:10.3390/ijms222010934)
Supplement: Supplementary file 1 [file ijms-22-10934-s001.zip › ijms-1391055-supplementary.pdf]

Table S1. Frequency distribution of 24 T cell receptor (TCR) Vβ families within different T cell subsets.

The table depicts frequency (%) of 24 studied TCR Vβ clones within conventional helper T cell (Th), cytotoxic T cell (Tc) and regulatory T cell (Tregs) subsets of healthy donors and the B-cell deficient patient. Individual results for three healthy donors (*C1*, *C2* and *C3*) are shown together with the mean value (*Mean*).

	**Vβ TCR**	**Th cells (CD3+CD4+FoxP3-)**					**Tc cells (CD3+CD8+)**					**Tregs (CD3+CD4+CD25HighFoxP3+)**				
No.		C1	C2	C3	Mean	Patient	C1	C2	C3	Mean	Patient	C1	C2	C3	Mean	Patient
1.	Vβ1	2.8	2.4	3.4	2.9	2	5.4	3.4	3	3.9	1.8	3.9	4.3	1	3.1	2.2
2.	Vβ2	6.2	7.4	11.1	8.2	6.3	4.1	2.3	4.7	3.7	16.7	3.1	6.8	10.8	6.9	10.0
3.	Vβ3	1.5	4.1	4.6	3.4	1.4	0.3	2.9	5.7	3.0	0.6	1.2	0	3.3	1.5	1.0
4.	Vβ4	2.2	2.2	2.1	2.2	1.8	0.5	1.5	1.4	1.1	0.2	1.8	1.6	1	1.5	1.8
5.	Vβ5.1	6.6	6	7.3	6.6	5.8	1.6	5.4	1.6	2.9	0.6	7.6	3.1	9.9	6.9	5.2
6.	Vβ5.2	0.9	1	0.7	0.9	0.6	0.4	0.6	0.3	0.4	0.1	2.3	2.3	0.5	1.7	0.4
7.	Vβ5.3	1.2	1.2	1.3	1.2	1.7	0.7	0.8	1	0.8	0.1	0	1.3	0.5	0.6	0.9
8.	Vβ7.1	1.9	2	2.3	2.1	1.5	1.4	2.5	3	2.3	1.4	1.2	1.3	2.7	1.7	2.5
9.	Vβ7.2	1.6	1.2	1.1	1.3	0.7	1.9	1.3	1.7	1.6	1.8	0.9	0	1.5	0.8	1.2
10.	Vβ8	4.9	3.8	4.7	4.5	2.8	2.1	2.9	6.5	3.8	1.8	8.4	0.9	1.9	3.7	3.0
11.	Vβ9	3.5	4	6.2	4.6	5.3	0.9	2.5	15.3	6.2	0.8	6	4.3	6.9	5.7	5.5
12.	Vβ11	0.8	0.8	1.3	1.0	0.7	0.4	1	0.9	0.8	0.1	0	0	1.4	0.5	2.8
13.	Vβ12	1.5	2.1	2.4	2.0	1.9	0.6	0.8	0.9	0.8	0.6	2.3	2.3	0.5	1.7	1.4
14.	Vβ13.1	4.1	4.3	3.6	4.0	3.4	1.1	3.4	3.2	2.6	3.9	4.7	2.8	3.4	3.6	3.9
15.	Vβ13.2	4.7	3	2.7	3.5	3.2	4.3	3.1	3.9	3.8	1.1	8	2.4	2.4	4.3	4.6
16.	Vβ13.6	1.7	1.7	1.4	1.6	1.8	0.5	0.9	0.8	0.7	0.4	2.8	1.9	1	1.9	2.3
17.	Vβ14	2.2	2.2	1.9	2.1	1.6	2.7	4	3.1	3.3	2.0	2.2	4.1	2.9	3.1	0.8
18.	Vβ16	1	0.7	1	0.9	0.6	0.7	2.9	0.8	1.5	0.5	2	2.2	0.9	1.7	0
19.	Vβ17	4.4	8.4	5.6	6.1	11.3	2.1	3.3	3	2.8	0.1	4	6.5	5.6	5.4	6.5
20.	Vβ18	0.3	0.4	0.9	0.5	0.3	0.6	0.3	0.5	0.5	1.4	0	1	0	0.3	0.4
21.	Vβ20	0	0	0.3	0.1	0	0	0	0.1	0	0	0	0	0.5	0.2	0
22.	Vβ21.3	2.4	1.9	2.7	2.3	3.2	1.1	3.1	1.4	1.9	2.9	1.9	0.7	1.6	1.4	3.1
23.	Vβ22	3	4.3	4	3.8	11.6	2.5	2.7	4.1	3.1	1.8	3.9	2.7	1.4	2.7	4.7
24.	Vβ23	0.6	0.6	0.7	0.6	0.8	0.6	1.2	0.8	0.9	7.4	1	1.4	0	0.8	1.4

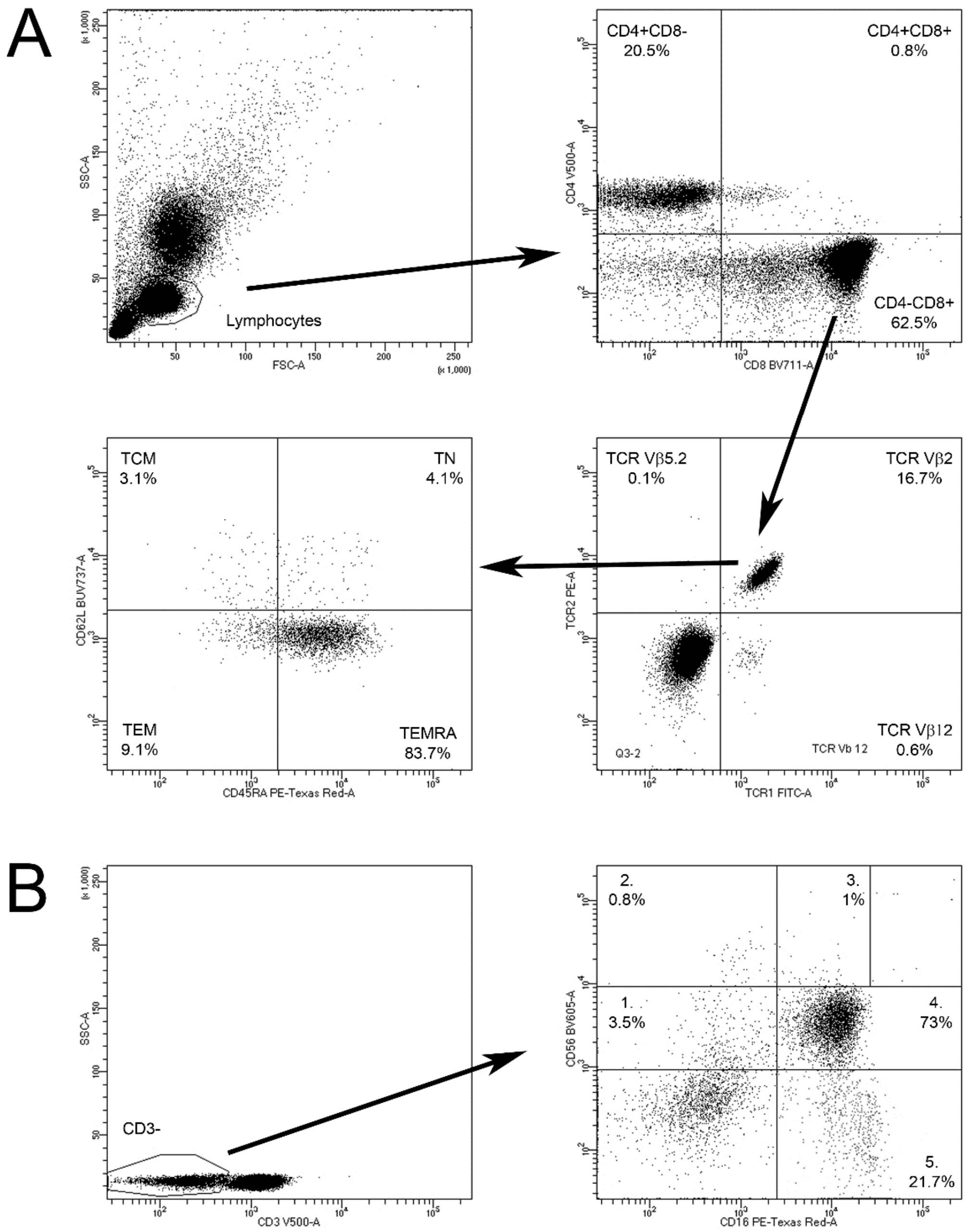

Figure S1. Gating strategy (A) depicts gating strategy for analysis of TCR Vβ2 cytotoxic T cells (Tc). First, lymphocytes were gated according to FSC and SSC values. Then, events from the later gate were plotted on CD4 vs. CD8 graph. Tc cells were identified as CD4^{-}CD8^{+} lymphocytes. Subsequently, events from Tc gate were plotted on TCR axes. Each sample was analyzed eight times with different set of anti-TCR antibodies to obtain the data for 24 TCR Vβ families. Then, each TCR Vβ family was analyzed separately for expression of various markers. The figure depicts as an example an identification of naive (TN), central memory (TCM), effector memory (TEM) and effector memory T cells that re-expressed CD45RA (TEMRA) within the TCR VβTc cell population of the patient. (B) Depicts gating strategy for identification of five NK cell subsets. First, CD3^{-} lymphocytes were gated. Then, the events from the latest gate were plotted on CD56 vs. CD16 graph. Subsequently, the following NK cell subsets were identified according to the intensity of CD56 and CD16 expression: (1) CD56^{dim}CD16^{-}; (2) CD56^{bright}CD16^{-}; (3) CD56^{bright}CD16^{dim}; (4) CD56^{dim}CD16$^{+;}$ and 5). CD56^{-}CD16^{+}.

Table S2. Characteristics of TCR Vb2+CD8+ T cells in the patient and healthy donors.

The table depicts frequency (%) of naïve (*T_N*), central memory (*T_{CM}*), effector memory (T_{EM}) and effector memory CD8$^+$ T cells that re-expressed CD45RA (T_{EMRA}) within TCR Vb2$^+$CD8+ T-cell population. In addition, distribution of cells expressing perforin (*P*), activation marker 4-1BB, IFN-γ and IL-10 is also shown for the studied TCR Vβ2$^+$CD8+ T-cell population. Each time results for the patient and healthy donors (HD) are shown. Individual results for three HD (*HD1*, *HD2* and *HD3*) are shown together with the mean value (*Mean*). The most striking differences between the patient and HD are marked in gray.

		TCR Vb2$^+$ CD8+ T cells				
	**Subpopulation**	HD1	HD2	HD3	Mean	Patient
Naive & memory	T_N [%]	20.8	55.9	43.7	40.1	4.1
	T_{CM} [%]	18.3	17.9	12.2	16.1	3.1
	T_{EM} [%]	25.5	20.1	35.9	27.2	9.1
	T_{EMRA} [%]	35.4	6.1	8.2	16.6	83.7
Perforin & 4-1BB	P+4-1BB- [%]	1.4	0	21.7	7.7	3.4
	P+4-1BB+ [%]	28.7	3.3	30	20.7	82.1
	P-4-1BB- [%]	8.6	8.1	23.8	13.5	0.4
	P-4-1BB+ [%]	61.3	88.6	24.5	58.1	14.1
IL-10 & IFN-g	IL10$^+$IFN-g$^-$ [%]	42.8	65.3	0.3	36.1	37.6
	IL10$^+$IFN- g$^+$ [%]	33.6	11.6	29.1	24.8	40.8
	IL10$^-$IFN- g$^-$ [%]	7.6	18.6	0.2	8.8	8.2
	IL10$^-$IFN- g$^+$ [%]	16	4.6	70.4	30.3	13.4

Table S3. Proportions of NK cell subsets and analysis of their cytotoxic potential.
The table depicts distribution of five NK cell subsets and expression of perforin (*P*) and granzyme A (*G*) in CD56dimCD16+, CD56brightCD16dim and CD56-CD16+ NK cell populations. Each time results for the patient and healthy donors (*HD*) are shown. Individual results for three HD (*HD1, HD2* and *HD3*) are shown together with the mean value (*Mean*). The most striking differences between the patient and HD are marked in gray.

		**NK cells**				
	**Subpopulation**	HD1	HD2	HD3	Mean	Patient
NK cell subsets	CD56dimCD16+ [%]	82.8	88.1	90.4	87.1	73
	CD56brightCD16-[%]	2.8	3.0	0.8	2.2	0.8
	CD56dimCD16- [%]	2.8	2.2	3	2.7	3.5
	CD56brightCD16dim [%]	4.2	1.4	0.5	2.0	1.0
	CD56-CD16+ [%]	7.4	5.3	5.3	6.0	21.7
$CD56^{dim}$ $CD16^{+}$	G+P- [%]	4.8	4.2	1.5	3.5	1.7
	G+P+ [%]	94	95.4	98.4	95.9	97.5
	G-P- [%]	1.0	0.1	0.1	0.4	0.2
	G- P+ [%]	0.2	0.3	0	0.2	0.6
$CD56^{bright}$ $CD16^{dim}$	G+P- [%]	53.1	38.1	12.9	34.7	20.5
	G+P+ [%]	28.7	38.2	87.1	51.3	52.3
	G-P- [%]	15.0	23.7	0	12.9	4.5
	G- P+ [%]	3.2	0	0	1.1	22.7
CD56- CD16+	G+P- [%]	48.0	49.8	36.4	44.7	83.4
	G+P+ [%]	38.1	28.7	53.7	40.2	15.6
	G-P- [%]	13.0	21.5	9.1	14.5	1.0
	G- P+ [%]	0.9	0	0.8	0.6	0
